# Bilayered, non-cross-linked collagen matrix for regeneration of facial defects after skin cancer removal: a new perspective for biomaterial-based tissue reconstruction

**DOI:** 10.1007/s12079-015-0313-7

**Published:** 2015-12-09

**Authors:** Shahram Ghanaati, Adorján Kovács, Mike Barbeck, Jonas Lorenz, Anna Teiler, Nader Sadeghi, Charles James Kirkpatrick, Robert Sader

**Affiliations:** 1 Frankfurt Orofacial Regenerative Medicine (FORM) Lab, Department for Oral, Cranio-Maxillofacial and Facial Plastic Surgery, Medical Center of the Goethe University Frankfurt, Frankfurt am Main, Germany; 2 Private Office, Nauheim, Germany; 3 Head and Neck Surgical Oncology, George Washington University, Washington, DC USA

**Keywords:** Inflammation, BCC, Skin cancer, Flaps, Mucograft, Soft tissue regeneration, Facial plastic surgery

## Abstract

Classically skin defects are covered by split thickness skin grafts or by means of local or regional skin flaps. In the presented case series for the first time a bilayered, non-crossed-linked collagen matrix has been used in an off-label fashion in order to reconstruct facial skin defects following different types of skin cancer resection. The material is of porcine origin and consists of a spongy and a compact layer. The ratio of the two layers is 1:3 in favour of the spongy layer. The aim of the study was to investigate the potential of this matrix for skin regeneration as an alternative to the standard techniques of skin grafts or flaps. Six patients between 39 and 83 years old were included in the study based on a therapeutic trial. The collagen matrix was used in seven defects involving the nose, eyelid, forehead- and posterior scalp regions, and ranging from 1,2 to 6 cm in diameter. Two different head and neck surgeons at two different institutions performed the operations. Each used a different technique in covering the wound following surgery, i.e. with and without a latex-based sheet under the pressure dressing. In three cases cylindrical biopsies were taken after 14 days. In all cases the biomaterial application was performed without any complication and no adverse effects were observed. Clinically, the collagen matrix contributed to a tension-free skin regeneration, independent of the wound dressing used. The newly regenerated skin showed strong similarity to the adjacent normal tissue both in quality and colour. Histological analysis indicated that the spongy layer replaced the defective connective tissue, by providing stepwise integration into the surrounding implantation bed, while the compact layer was infiltrated by mononuclear cells and contributed to its epithelialization by means of a „conductive“process from the surrounding epithelial cells. The clinical and histological data demonstrate that the collagen bilayered matrix used in this series contributes to a „Guided-Integrative-Regeneration-Process“, which still needs to be further understood. The biomimetic nature of this material seems to contribute to physiological matrix remodelling, which probably involves other matricellular proteins essential for soft tissue regeneration. A deeper understanding of the mechanism, involved in the tissue integration of this material and its contribution to soft tissue regeneration based on the direct and indirect effect of matricellular proteins could open new therapeutic avenues for biomaterial-based soft tissue regeneration as an alternative to traditional flap-based plastic surgery.

## Introduction

Currently the surgical treatment of extraorally located benign or malignant tumors of the facial skin involve tumor resection and defect reconstruction either with free skin grafts or regional flaps, which are moved to the defect by means of various flap mobilization techniques [Sandu et al. 2012; Zhao et al. 2012; Rogers-Vizena et al. 2015]. All these techniques are associated with mobilization of neighbouring soft tissue in order to achieve tension-free wound closure. Accordingly, in relation to the defect size large incision and mobilization of the neighbouring soft tissue is needed in many cases. The latter contributes to large skin wounds, associated with the risk of damaging other important tissues for facial function, such as vessels and nerves [Rogers-Vizena et al. 2015]. Large surgical wounds carry the additional risk of infection and even skin necrosis if the accurate dimensions of the flap base and flap length are not respected [Sameem et al. 2012]. There are some regions in the face, such as the nose and forehead, where skin tissue mobilization for flap extent is compromised. Consequently, the dangers of raising the flap are increased. Thus, techniques to reduce the amount of mobilization to a minimum and at the same time permit tension-free closing of the wound are of high interest to reduce morbidity of such surgical interventions. Aesthetic considerations are also justified in the face, because larger incisions bear the risk of greater visibility.

In recent years a bilayered porcine collagen matrix (BPCM) consisting of a membranous as well as a spongy component was introduced as a reliable material for regeneration of oral soft tissue [Sanz et al. 2009; Ghanaati et al. 2011; Jepsen et al. 2013]. Clinicians have been able to demonstrate the suitability of this material to improve significantly the soft tissue condition within the oral cavity around dental soft tissue defects, such as its integration in vestibuloblasty procedures [Sanz et al. 2009; Herford et al. 2010; Jepsen et al. 2013; Schmitt et al. 2013; Schmitt et al. 2015]. Vestibuloplasty surgery involves the formation of a mucosal flap to re-induce the attached keratinized gingiva [Schmitt et al. 2013; Schmitt et al. 2015]. Collagen membranes/matrices are used in this approach to cover the tissue level below the oral mucosa after its mobilization [Cardaropoli et al. 2012]. Accordingly, these materials are placed on the periostium or the perio-myofiber tissue level and their outer edges are fixed in close proximity to the neighbouring oral mucosa. The spongy part of the material integrates into the underlying tissue, while its oral cavity facing the membranous surface is epithelialized by the neighbouring mucosal epidermal layer. This process contributes ideally to the generation of a new keratizined gingival tissue in regions in which a vestibuloplasty is necessary [Cardaropoli et al. 2012]. Thus, research and development in oro-maxillofacial surgery have culminated in the establishment of animal-derived membranes and matrices for regeneration of soft tissue [Gottlow et al. 1984; Eickholz et al. 2007; Pretzl et al. 2008]. However, until now the mechanism involved in the integration of the BPCM into the oral soft tissue is not elucidated.

Considering similarities (i) in the surgical procedure relying on flap techniques as well as (ii) similarities in anatomy and histology between the oral mucosa and facial skin (Table 1), we hypothesized that the bilayered porcine-based collagen matrix (BPCM) might also be useful in extraoral regions to regenerate skin. Thus, in the light of recent developments in biomaterial research, especially for intraoral soft tissue, we considered whether the same approach could be used for defect reconstruction in extraoral regions such as facial skin.

**Table 1:** Comparison of the histological composition of the human keratinized gingiva and skin

Stratification	Keratinized Gingiva	Skin
Underlying tissue layers	*Lamina propria*	*Dermis* and *Subcutis*
Proliferative cell layer/Cell-reservoir	*Stratum basale*	*Stratum basale*
Cell maturation	*Stratum spinosum* *Stratum granulosum*	*Stratum spinosum* *Stratum granulosum*
Superficial cell layer/Mechanical protection and bacterial barrier	*Stratum corneum*	*Stratum corneum*

Besides the aformentioned BPCM other collagen membranes/matrices are on the market, which differ in source of collagen and manufacturing processes. Interestingly, despite the fact that all are collagen membranes/matrices they induce different cellular inflammatory responses after implantation, when applied subcutaneously in experimental animals. After a primary neutrophil-driven cellular response they induce either a mononuclear/macrophage- [Ghanaati et al. 2011; Ghanaati 2012] or a multinucleated-based [Barbeck et al. 2014a; Barbeck et al. 2014b] cellular response. The mononuclear cell response contributes to the integration of the collagen membrane/matrix within its host tissue, combined with a mild vascularization of the implantation bed and integration of the respective collagen membranes/matrices [Ghanaati et al. 2011; Ghanaati 2012].

Multinucleated cells - also known as foreign body giant cells - are specialized immune cells generated via macrophage fusion to eliminate foreign antigens via e.g. proteolytic degradation mechanisms [Anderson 2001]. The induction of these cells is associated with an increased vascularity of the implantation bed and results in material degradation and the breakdown of its structural integrity [Barbeck et al. 2014a; Barbeck et al. 2014b]. The latter scenario might result in an insufficient premature connective tissue ingrowth into the implantation bed and contribute to the initiation of scar tissue. Accordingly, materials for the generation of tissue volume should not undergo premature breakdown linked to the formation of multinucleated giant cells [Ghanaati et al. 2011; Ghanaati 2012; Barbeck et al. 2014a; Barbeck et al. 2014b]. Previously published histological data from our group demonstrated the integration of the above-mentioned BPCM within mouse subcutaneous tissue and human gingival tissue. In both species no material breakdown and almost no occurrence of multinucleated giant cells were observed [Ghanaati et al. 2011].

Considering the above-mentioned variable cellular reaction to several “nature-derived” materials, it becomes obvious that there might be fundamental differences in the way these materials initially interact with the host inflammatory cascade, and on a deeper level, by the extracellular matrix components involved in tissue remodelling [Bornstein and Sage 2002]. Matricellular proteins have been shown to be involved in the process of the foreign body reaction [Kyriakides and Bornstein 2003], especially after biomaterial implantation [Morris and Kyriakides 2014]. In the present clinical case series patients with malignant skin tumors were treated solely by means of the bilayered porcine collagen matrix. The aim was to address clinically and histologically the suitability of the matrix to regenerate skin after full thickness skin excision. Considering the important role of matricellular proteins in tissue-biomaterial-interaction, this paper should stimulate further research into the mechanisms by which matricellular proteins are involved in tissue-biomaterial-interactions.

## Materials and methods

### Material characterization

Mucograft® (Geistlich Pharma AG Wolhusen, Suisse) is a CE-marked and FDA-approved three-dimensional matrix registered at the time of this study for intraoral application. It is made from pure collagen obtained by a proprietary standardized manufacturing process and sterilized by gamma irradiation. This bilayered porcine collagen matrix (BPCM) consists of collagen type I and type III of porcine origin without further cross-linking or chemical treatment. The matrix consists of a bilayered structure with a thin, smooth and low-porosity compact layer (CL) in combination with a more porous three-dimensional spongy layer (SL). The CL allows suturing to the mucosal margins. The SL is turned towards the bone defect and/or soft tissue and thereby enables tissue ingrowth to support wound healing.

### Patient collection, operation and postoperative care

In the presented case series six patients with seven lesions within the forehead, the inner lid angle, the nasal regions, the posterior scalp and the pre-auricular regions were treated with Geistlich Mucograft (BPCM) in an off-label fashion after tumor resection based on a therapeutic trial. The patients suffered from sclerodermia, actinic keratosis, basal cell carcinoma and squamous cell carcinoma. An overview of the patients as well as the specifications of the lesions are given in Table 2. Three of the patients were treated in a private practice by one of the first authors (AK), while the remaining 3 patients were treated at the Department of Oral, Cranio-Maxillofacial and Facial Plastic Surgery in Frankfurt by the other first author (SG). Prior to surgery the patients were informed about the required surgical procedure, possible adverse effects and the application of the bi-layered porcine collagen matrix (Geistlich Mucograft®, Geistlich Pharma AG, Wolhusen, Switzerland) and gave written consent to agree with the material’s off-label use as well as publication of their faces without eye coverage. All patients gave written consent for regular follow-ups, which comprised the clinical and digital assessment of the wound healing process. The three patients operated by (AK) additionally agreed to be biopsied at day 14 after the operation.

**Table 2:** Patients

Patient	sex	Age (a)	Localisation of defect	Defect size (mm)	Histopathology
1	f	39	Right inner lid angle	12x12x3	Spiradenoma
2	m	72	Left alar wing	14x13x3	Basal cell carcinoma
3	m	69	forehead	20x20x4	Squamous cell carcinoma
4	m	74	Nasal bridge	10x8x3	Actinic keratosis
			Left nasal wing	16x10x3	Basal cell carcinoma
5	m	49	Left occiput	36x40x14	Spinocellular carcinoma
6	m	83	Right pre-auricular	60x50x16	Squamous cell carcinoma

After the tumor resection the defects had a diameter range from 1,2–6 cm. In all patients complete resection with wide margins and reconstruction with BPCM was planned. The spongy part of BPCM was placed onto the wound surface, while the membranous layer was “air” exposed, respectively. BPCM was sutured into the surrounding tissue of the defect and covered by a conventional pressure dressing in the cases treated by AK. In cases operated by SG a latex-based sheet obtained from a piece of sterile surgical glove was used as a cover underneath the pressure dressing. In two of these three cases a multilayer technique was employed to increase the vertical dimension of the defect. In one of the two cases, multiple 3 mm in diameter punches were taken from the BPCM to increase the vertical growth of the patient’s native connective tissue. In all cases the dressing was changed at day three postoperatively, and sutures were removed on day six. Both parties used their wound dressing until first visible epithelialisation of the wound. Patients were followed up for a duration of eight weeks on a nearly weekly basis extended to longer follow-up periods for up to one year after the surgical procedure.

### Histological analysis of the biopsies

#### Tissue preparation for histochemistry

From patients 1–3 cylindrical samples of 3 mm in diameter were taken from the central region of the reconstructed region at day 14 after implantation. The samples were subsequently fixed in 4 % buffered formalin for 24 h, dehydrated in a series of alcohols and xylene and embedded in paraffin. The tissues were cut along the longitudinal axis in sections of 3–4 μm thickness. Sections were deparaffinized and stained for histology as previously described [Ghanaati et al. 2011; Ghanaati 2012; Barbeck et al. 2014a; Barbeck et al. 2014b]. The first slide was stained with standard Mayer’s hematoxylin and eosin (H&E). The second and third slides were stained with Azan and Sirius red, respectively. Moreover, four further slides of each explant were used for immunohistochemical detection of macrophages (anti-human CD68 antibody, Clone: EBM11, dilution: 1:300, Dako, Denmark) and Tartrate-Resistant-Acid-Phosphatase (TRAP)-expressing cells (TRAP Antibody (K-17), dilution: 1:300, Santa Cruz Biotechnology, US) within the implantion bed of the BPCM as also previously published [Ghanaati et al. 2011; Ghanaati 2012; Barbeck et al. 2014a; Barbeck et al. 2014b, Ghanaati et al. 2014; Lorenz et al. 2014]. Thereby, two slides were used as negative controls without incubation with the first antibody.

#### Morphological evaluation of the biomaterial-specific inflammatory response

The tissue reaction and the biomaterial-specific inflammatory response were evaluated histologically at the FORM-Lab (Research Laboratory of Department of Oral, Cranio-Maxillofacial and Facial Plastic Surgery, Medical Center of the Goethe University Frankfurt, Germany) using a Nikon ECLIPSE 80i microscope (Nikon, Tokyo, Japan) by SG and MB. Microphotographs of the analyzed biopsies were recorded with a connected digital camera DS-Fi1 together with a Nikon digital sight control unit (Nikon, Tokyo, Japan). A qualitative histological analysis was performed by SG and MB to evaluate the tissue response, as well as degradation and integration behavior of both implanted biomaterials. Furthermore, cells participating in the process of biomaterial integration, degradation, vascularization and possible adverse reactions such as fibrotic encapsulation or necrosis were identified.

## Results

### General observation

All patients survived the treatment and did not show any sign of adverse effects during the healing period and therafter until the present time. No material loss or abnormal drying was observed during the healing period of BPCM. In all cases a complete healing was observed without any sign of material remnants at the defect-scar-interface. In all cases a satisfactory condition for the patients could be achieved. Patients did not report any necessary adjustment to their everyday life during the healing process of their wound as treated by BPCM. None of the patients treated with BPCM showed any sign of premature scar formation either in close proximity or in the more distant neighbourhood of the defect, although it seems that defect size above 2 cm may contribute to a shiny look of the skin. From the cosmetic point of view all patients were satisfied with the results. The results of each patient will be discussed below separately.

### Specific observation

#### Case 1

**Fig. 1:**
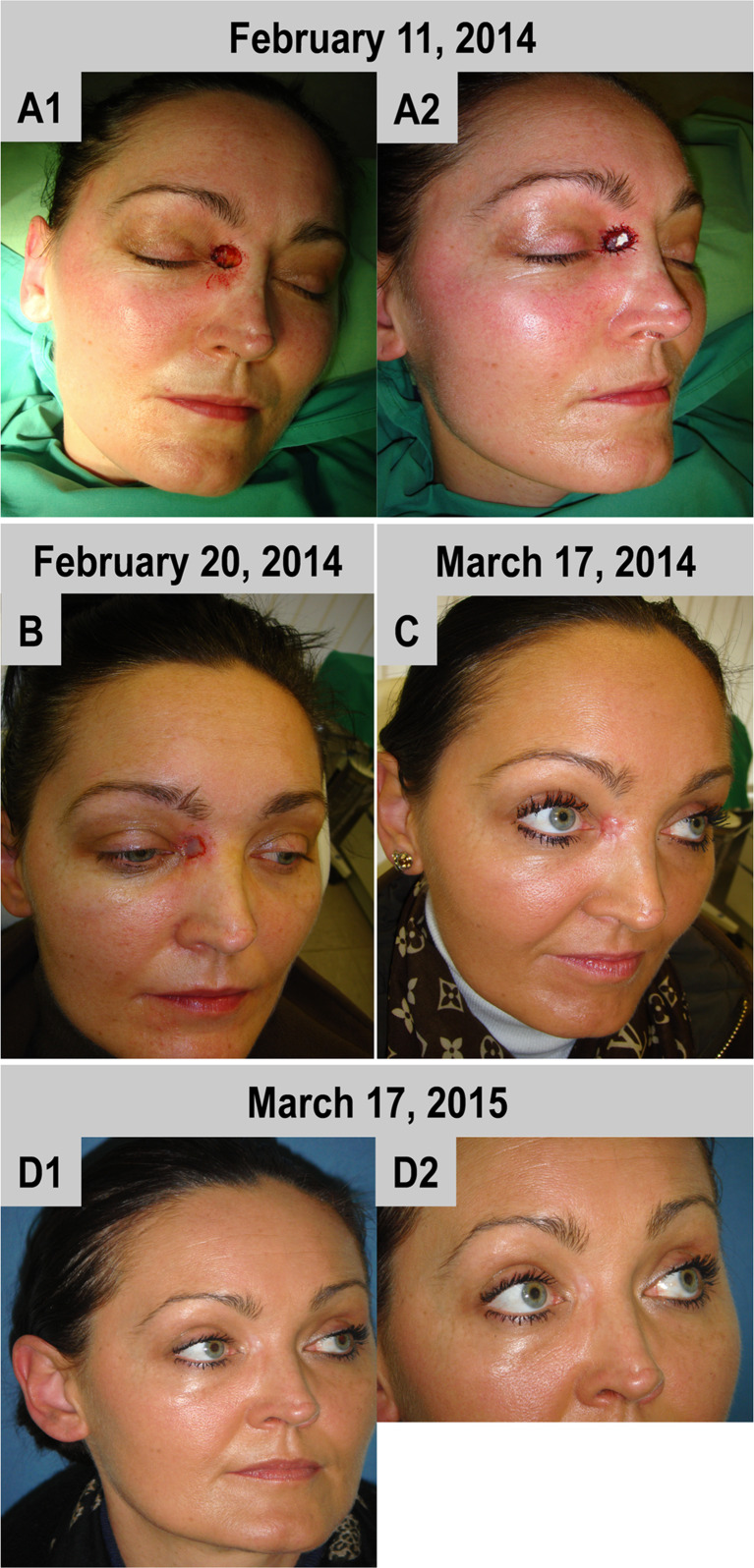
BPCM-based tissue regeneration of an eyelid defect of a 39 year-old female patient. **a**-**c** illustrate the soft tissue regeneration after six weeks, while **d** shows the result after one year

The defect at the inner lid angle was able to be covered by one layer of BPCM. A good adaptation of the BPCM to the wound edges was achieved and subsequently observed (Fig 1). Wound closure with a thin epidermis layer commenced at day 10 after implantation (Fig. 1b) and initial wound closure was reached within a period of 14 days (data not shown). A tension-free tissue regeneration within the area of the former defect was completed within a time period of five weeks (Fig. 1c). No tension in the neighbouring regions or damage to eyelid dynamics, such as opening and closure, could be observed (Fig. 1c). After five weeks regeneration time the newly formed skin in the original defect region was more reddish and elevated when compared to the neighbouring skin tissue (Fig. 1c). The newly regenerated skin, however, flattened with time and adapted to the neighbouring skin level (Fig. 1d). Accordingly, after even one year no scar formation or anatomic distortion was observed within the area of the non-cross-linked collagen matrix-based regenerated skin (Fig. 1d).

#### Case 2

**Fig. 2:**
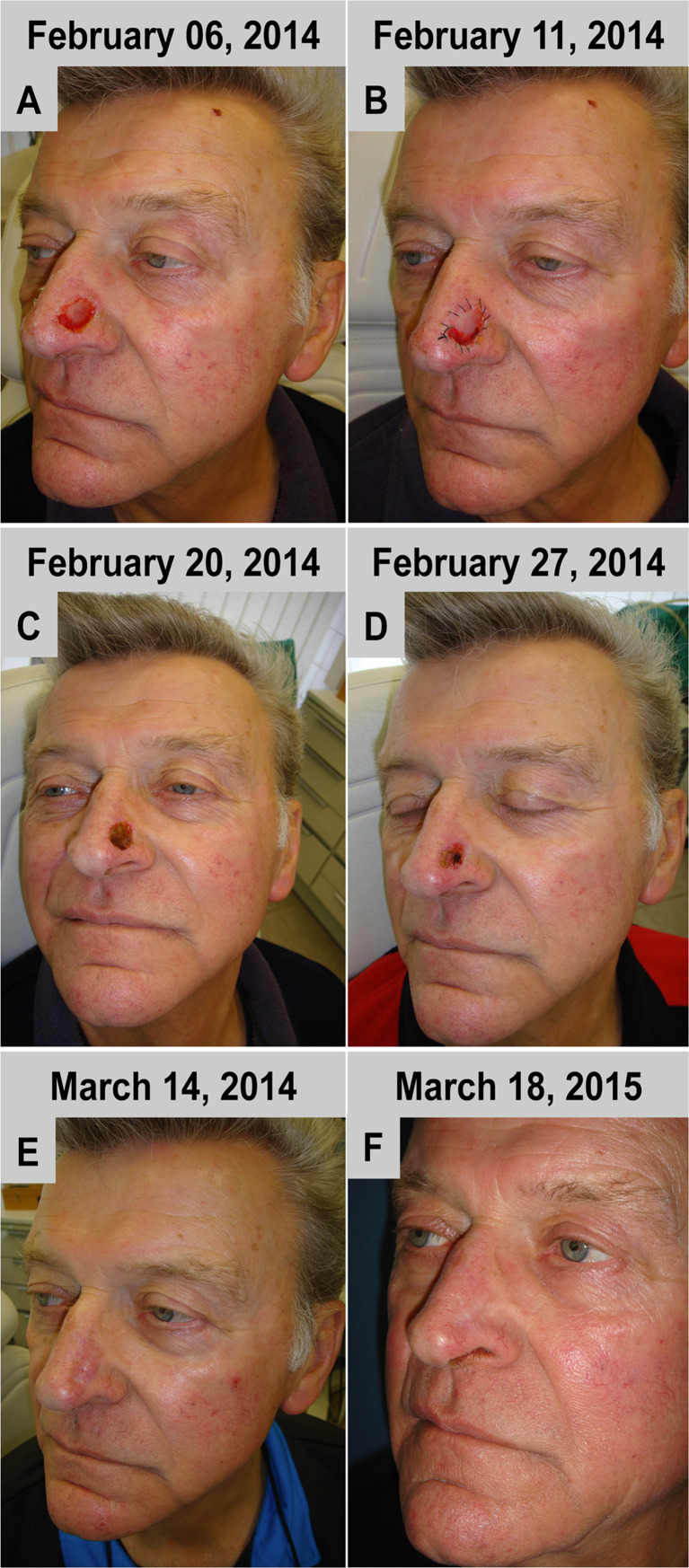
BPCM-based tissue regeneration of a nose region of a 72 year-old male patient. **a**-**e** are representative of the state of soft tissue regeneration after six weeks, while **f** shows the result after one year

The defect above the left nasal ala was completely covered by one layer of the BPCM without any complications (Fig. 2a). The BPCM edges were able to be stitched without complication to the tissue surrounding the defect (Fig. 2b). The spongy layer found a good adaptation with the wound surface. Also here the initial wound closure was reached within a time frame of 14 days after material implantation (Fig. 2c). Tissue regeneration within the area of the former defect was completed in five weeks without any tension of the neighbouring regions or without harming alar dynamics, i.e. its shape was retained during inspiration and expiration (Fig. 2). In the augmented area a more elevated and reddish regenerated skin area was evident when compared to the neighbouring area (Fig. 2e). However, the newly regenerated skin flattened with time and adapted to the neighbouring skin level (Fig. 2f). After one year no scar formation or anatomic abnormality was observed within the area of the BPCM-based regenerated skin (Fig. 2f).

#### Case 3

**Fig. 3:**
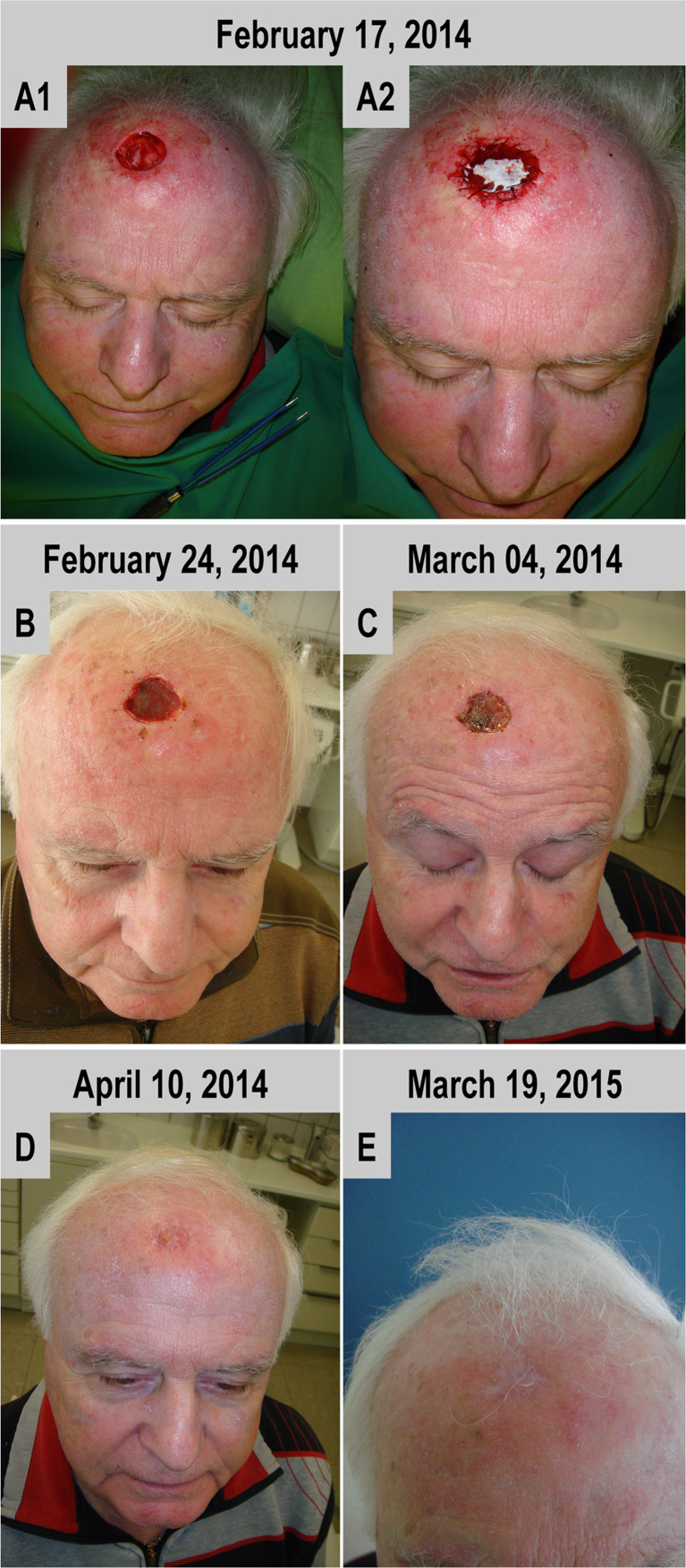
BPCM-based tissue regeneration of a forhead region of a 69 year-old male patient. **a**-**d** shows the soft tissue regeneration at six weeks, while **e**) gives the result after one year

A single layer of BPCM was sufficient to cover without any complications the defect in the forehead region (Fig. 3a). Comparable to the eyelid and nose tissue region, the matrix edges could be stitched with the surrounding tissue without complication (Fig. 3b). The spongy layer found a good adaptation with the wound surface. Initial wound closure was reached within a time frame of three weeks (Fig. 3c). Tissue regeneration within the area of the former defect was completed within a time period of seven weeks, without any tension of the neighbouring regions or without harming forehead dynamics, i.e. in its shape during activation of the mimic muscles in that region (Fig. 3). Similar to cases 1 and 2, within the forehead skin an elevated reddish regenerated skin area in comparison to the neighbouring area could be found (Fig. 3d). However, as was seen with the above-mentioned cases, the newly regenerated skin flattened with time and adapted to the neighbouring skin level (Fig. 3f). After one year no scar formation or anatomic lesion was observed within the area of the BCM-based regenerated skin, although a smooth glossy appearance remained. The latter might be related to a re-epithelization process (Fig. 3e).

#### Case 4

**Fig. 4:**
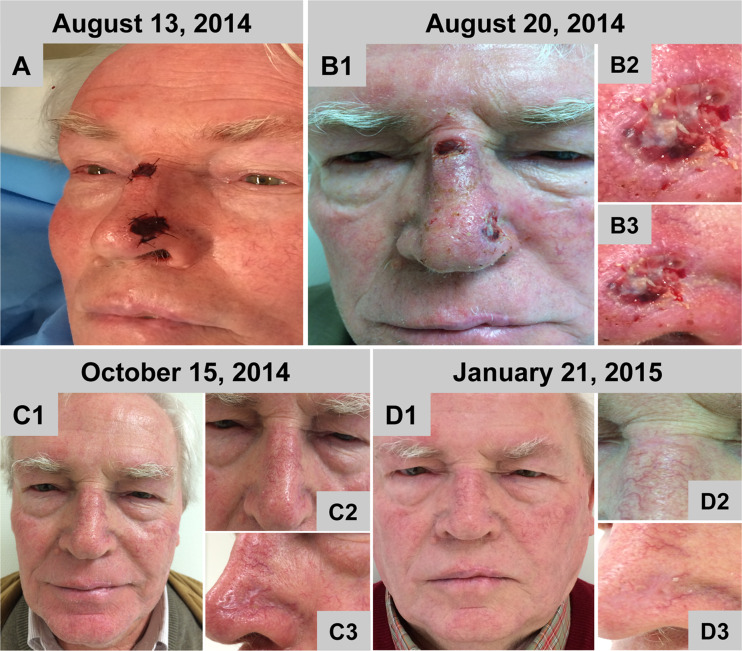
BPCM-based tissue regeneration of two nose defects of a 74 year-old male patient. **a**-**d** shows the soft tissue regeneration after six weeks, while **e** shows the result after one year. **a**-**c** shows the soft tissue regeneration at eight weeks. **d** shows the results after four months

Both defects on the nose could be completely covered by one layer of BPCM without any complications (Fig. 4a). In both regions, the matrix edges were easily stitched to the surrounding tissue. The spongy layer was well adapted to the wound surface. At both sites the initial wound closure could be reached and began seven days after material implantation (Fig. 4b). At the first wound dressing change the wounds had an exsudative character. Tissue regeneration within the area of the former defects was completed within a time period of six to eight weeks without any tension in the neighbouring regions or without harming the alar region as well as the medial region of the nose (Fig. 4). As reported for case 1–3, similar findings concerning the elevated reddish regenerated skin area in comparison to the neighbouring area could also be found at both nasal regions covered by BPCM (Fig. 4 1-C3). However, the newly regenerated skin flattened with time and adapted to the neighbouring skin level (Fig. 4 D1-D3). As early as four months later no anatomic deformity was observed within the area of the BPCM-based regenerated skin (Fig. 4 D1-D3). The regenerated skin promoted by the BPCM implantation appeared very similar to that of the neighbouring tissue (Fig. 4 D1-D3).

#### Case 5

**Fig. 5:**
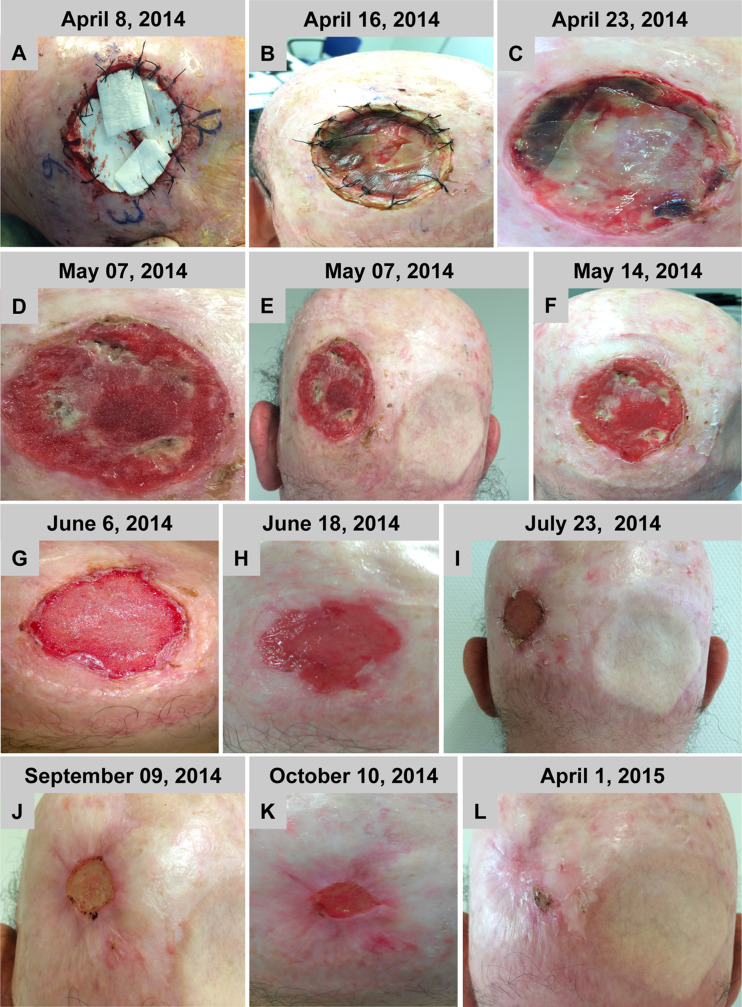
BPCM-based tissue regeneration of a posterior scalp defect of a 49 year-old male patient. **a**-**k** shows the soft tissue regeneration at six months, while **l** shows the result after one year

The defect on the posterior scalp could be completely covered by four sheets of the non-cross-linked collagen matrix, building a two-layer reconstruction. Thus, a multilayer reconstruction of the defect by means of the BPCM could be performed easily (Fig. 5a-c). It was possible to stitch the BPCM edges with the defect surrounding tissue. There was no technical problem in placing two matrices on top of each other, as the spongy layer of the superficial matrix found easy adaptation to the compact layer of the underlying one. Both layers adapted without complication to the wound surface (Fig. 5b). At the first wound dressing change the wound had an exsudative character and during the first 2–3 weeks after BPCM implantation the matrices gradually flattened to the deepest region of the wound (Fig. 5b and c). However, after that time period a vertical growth was observed within the defect area (Fig. 5d). It appeared as if the vertical growth stopped at the level of the neighbouring epithelial tissue (Fig. 5d-f). Accordingly, after reaching the vertical dimension of the neighbouring tissue horizontal tissue growth with new keratinized epithelium took place (Fig. 5g-k). The entire healing process took about 6–8 months. Following complete wound coverage (Fig. 5 l) the regenerated skin region showed similar morphology and appearance to the neighbouring tissue (Fig. 5 l). The patient was satisfied with the treatment with the here used BPCM, as he reported a prolonged hospital stay and high morbidity related to the skin graft healing after tumor resection of his right posterior scalp (Fig. 5e, i, and l).

#### Case 6

**Fig. 6:**
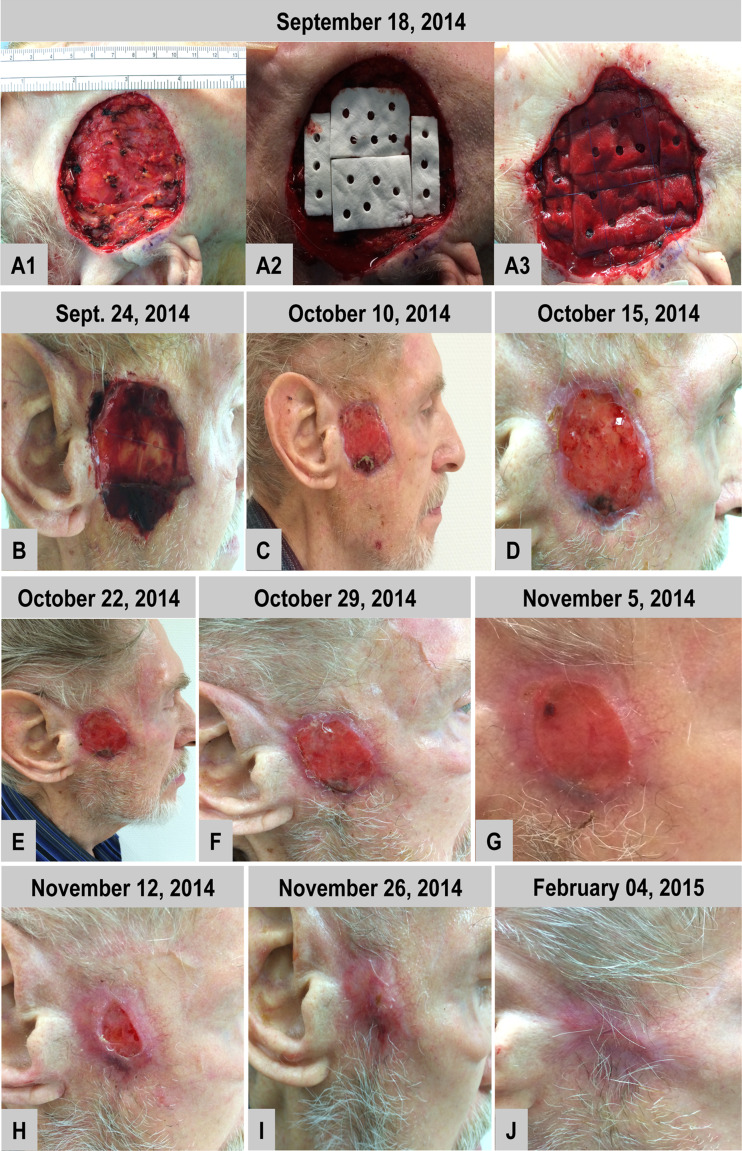
BPCM-based tissue regeneration of a preauricular region of a 83 year-old male patient. **a**-**l** illustrates the soft tissue regeneration at eight weeks, while **j** shows the result after five months

The defect in the pre-auricular region was successfully covered by three sheets of the BPCM, which were all punched before implantation. Thus, a reconstruction of the defect by means of the collagen matrix could be performed easily (Fig. 6 A2 and A3). It was possible to gently pull the surrounding tissue over the collagen matrix edges to reduce the size of the defect (Fig. 6 A3). Already after six days the defect had reached more and less the same vertical dimension as that of the surrounding tissue. Moreover, an exsudative wound was observed during the first wound dressing change. Following this, horizontal wound closure started (Fig 6 b-j) and tissue regeneration was completed in ten weeks, without giving any tension in the neighbouring regions (Fig. 6). In line with the clinical findings of the other cases an elevated reddish regenerated skin area was observed (Fig. 6j). The newly regenerated skin flattened with time and adapted to the neighbouring skin level (Fig. 6j). Six months later no deformity was observed within the area of the BPCM-based regenerated skin (Fig. 6j), which gave a functional and aesthetically acceptable result when compared with the neighbouring tissue (Fig. 6j).

### Histological results of the biopsies

**Fig. 7:**
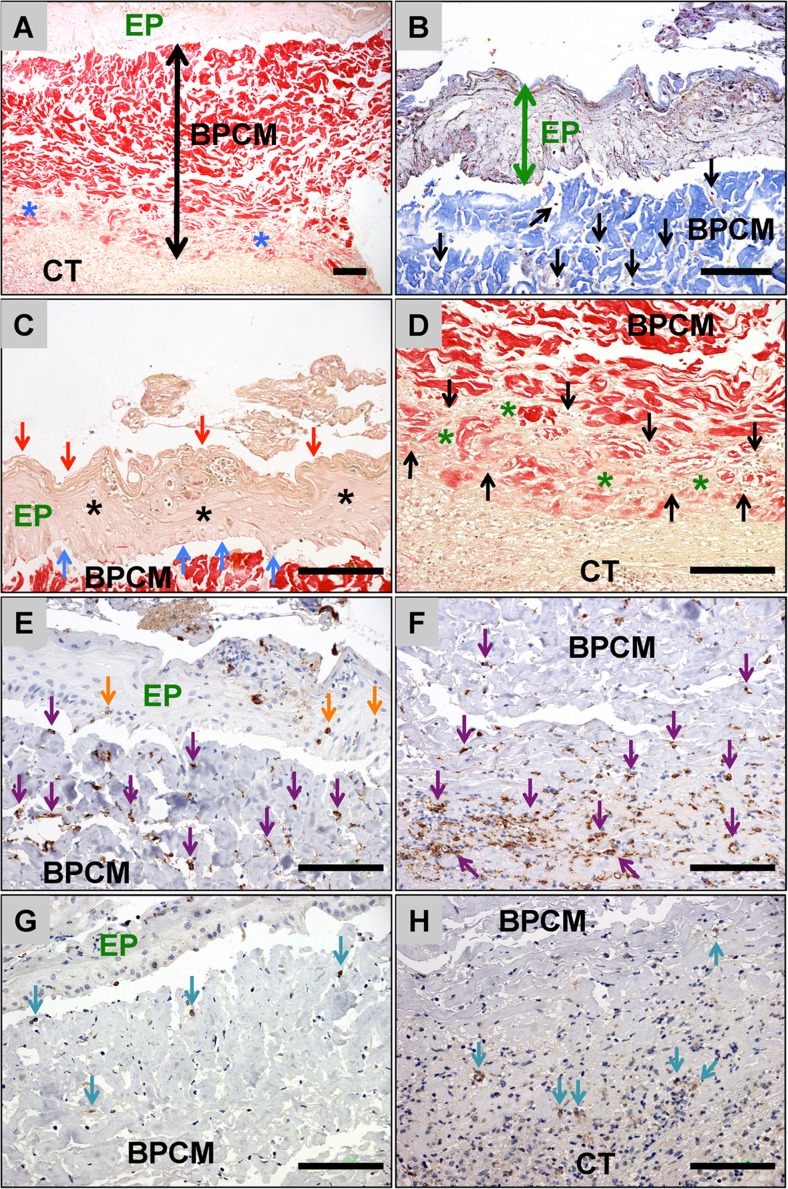
Exemparily histology of BPCM-based tissue regeneration of small-sized skin defects. a Overview of the implant area of CM (double arrows), which was placed above the subepidermal connective tissue (CT). Tissue ingrowth was seen at the spongy matrix layer (*blue asterisks*) adjacent to the wound base, while the upper compact layer was covered by regenerated epidermis (EP) (Sirius-staining, 100× magnification, scale bar =100 μm). **b** The spongy matrix layer was invaded only by single mononuclear cells (*black arrows*) and this matrix layer was directly contiguous to the regenerated epidermis (*EP*, *double arrow*) (Azan-staining, 200× magnification, scale bar =100 μm). **c** The epidermal layer (EP) covering the compact matrix layer (BPCM) showed a multi-layered cellular structure including a basal cell layer (*blue arrows*), the *Stratum granulosum*/*spinosum* (*black asterisks*) and the *Stratum corneum* (*red arrows*) (Sirius-staining, 200× magnification, scale bar =100 μm). **d** The outer region of the spongy layer of the BPCM covering the wound base (connective tissue, CT) was invaded by connective tissue matrix (*green asterisks*) and mononuclear cells (*black arrows*), while the central region was not integrated at this time point (Sirius-staining, 200× magnification, scale bar =100 μm). **e** and **f** The mononuclear cells that have invaded both the compact **e** and the spongy **f** matrix layers of the BPCM were identified mainly as macrophages (*purple arrows*). The immunohistochemical detection also showed the presence of cells of macrophage lineage within the covering epidermal layer (*EP*), indicating the complete regeneration of this tissue (CD68 immunohistochemical staining, 200× magnification, scale bars =100 μm). **g** and **h** The detection of TRAP showed that only a minority of the mononuclear cells that invaded the compact g and the spongy h layers of the BPCM showed signs of expression (TRAP immunohistochemistry, 200× magnification, scale bars =100 μm)

**Fig. 8:**
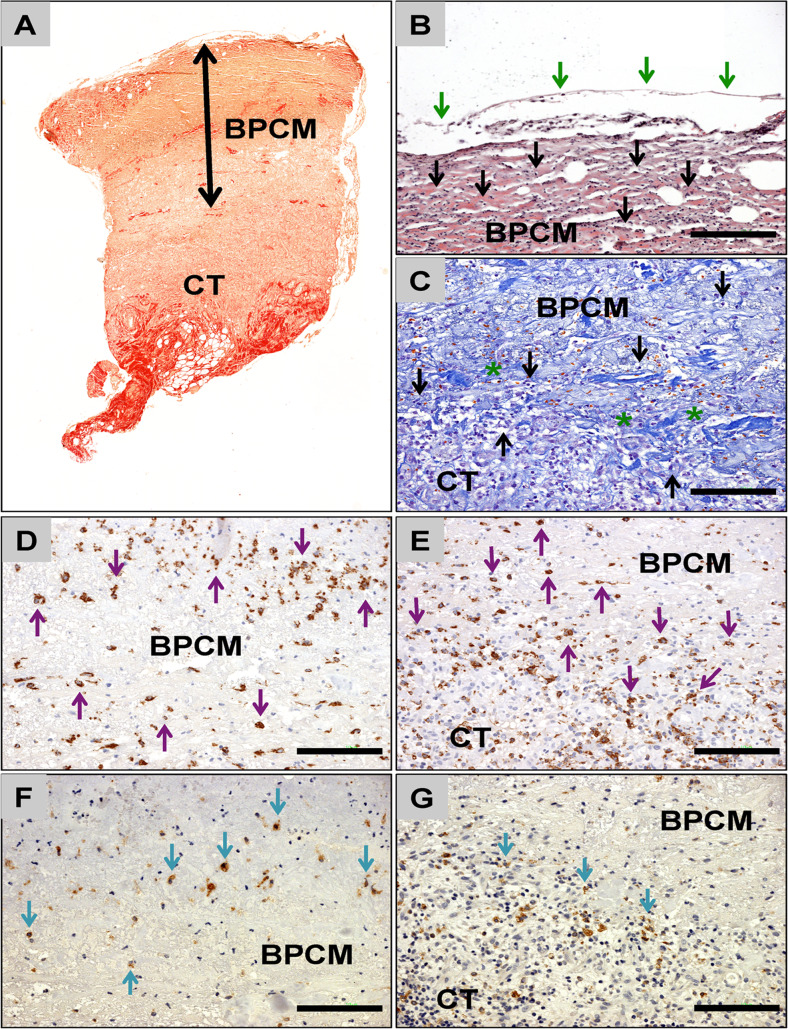
Exemparily histology of BPCM-based tissue regeneration of a medium-sized skin defect. **a** Overview of the implant area of BPCM (*double arrows*) implanted above the connective tissue (CT) of the wound base (Sirius-staining, „*total scan*“100× magnification). **b** The compact layer of the BPCM was covered by a thin and continuous layer of epidermal cells (*green arrows*). Mononuclear cells (*black arrows*) have invaded this material layer at this time point (HE-staining, 200× magnification, scale bar =100 μm). **c** The material of the spongy layer of the CM adjacent to the connective tissue of the wound ground (CT) was integrated into a connective tissue (asterisks) containing high numbers of mononuclear cells (black arrows) (Azan-staining, 200× magnification, scale bar =100 μm). **d - g** The CD-68 detection showed that most of the cells that invaded the compact **d** and the spongy **e** layer of the BPCM were macrophages (*purple arrows*). Only a few of the cells that have invaded the compact **f** and the spongy **g** components of the CM showed signs of TRAP-expression (**d** and **e**: CD68 immunohistochemistry, **f** and **g**: TRAP immunohistochemical staining, 200× magnification, scale bars =100 μm)

Figure 7 shows exemplary histological images of the integration behavior and the tissue reaction of/to the BPCM after its application for small-sized skin defects, while Fig. 8 shows images after the BPCM application in the case of medium-sized defects.

The histological analysis revealed that BPCM was detectable within the defect areas at day 14 after its application without evidence of material disintegration (Figs. 7a and 8a). The compact layer, which was adapted to the outer border of the defects in both cases only allowed the invasion of single mononuclear cells. No ingrowth of extracellular connective tissue elements was observed in this layer (Figs. 7a and 8a). Interestingly, in case of the small-sized skin defects this matrix layer was covered by a “complete“epidermal layer, charaterized by its multi-layered cell structure including the basal cell layer, the *stratum granulosum*/*spinosum* and the *stratum corneum* within the central defect area (Fig. 7b and c). In the case of the medium-sized defects histological analysis revealed that the compact collagen matrix layer was covered by a thin epidermal layer of 1–2 cell layers within the central defect area (Fig. 8b).

The outer region of the spongy matrix layer, i.e. the wound-material-interface was integrated in a cell-rich connective tissue involving only mononuclear cells (Figs. 7d and 8c). No histological signs of a severe inflammatory tissue response to this matrix layer were found. However, the central regions of the collagen matrix were free of cells or matrix elements at this time point (Fig. 7a and 8a).

Additionally, immunohistochemistry revealed that all cells invading both the compact (Figs. 7e and 8d) and the spongy matrix layers (Fig. 7f and 8e) were macrophages. Immunohistochemistry also showed that in the case of both layers only a minority of the invaded cells expressed TRAP (Figs. 7g, h, and 8f and g). No multinucleated giant cells were observed in both analyzed implantation beds.

## Discussion

In the presented clinical case series a bilayered collagen matrix (BPCM), commercially available for regeneration of oral tissue, was successfully applied in a dry state for skin regeneration in full thickness skin defects after skin cancer excision. The application of BPCM was found to be technically feasible in the setting of a private practice as well as a hospital. The wound healing course was uneventful in all patients and resulted in normal appearing skin in terms of coloration, structure, and consistency without clinical evidence of scaring or anatomic deformity. These findings are different than those observed in cases of open wound healing, as in the latter case a granulation tissue arises, which inevitably results in a contracted scar tissue.

The assessment of the first three cases perfomed by AK showed that BPCM is a suitable matrix for regeneration of small and middle-sized defects in nasal, eyelid and forehead regions. The clinical assessment, together with the digital images showed a more or less complete *restitutio ad integrum* as the result of BPCM application. The data reveal that all defects could be completely regenerated within a time frame of 4–6 weeks. It seems that the use of a conventional occlusive dressing with Melolin® as a non-adhesive dressing and Fixomull®-Stretch might be sufficient to cover the regenerated site after operation for 6 days until removal of the stitches. Clinical examination as well as the analysis of the digital images showed that in each case a reddish and elevated skin region could be observed after complete wound closure. This, however, flattened with time and thus the regenerated region seemed to adapt to the neighbouring tissue without any signs of deformity and scaring or tension in regard to the surrounding tissue. The cellular mechanisms responsible for this adaptation still remain to be elucidated. According to the presented spectrum of cases BPCM can be used for both young and elderly outpatients with tumors in aesthetically sensitive regions. The assessment of the remaining cases performed by SG showed that with the non cross-linked collagen matrix multiple defects also in patients with a complex medical history can be successfully regenerated. Thus, in relation to the defect size one regular sheet of BPCM sized 3 × 2 cm can be used for more than one defect within the face at one time point, improving time management in addition to the financial benefits for the patients.

In all cases the wounds at day six after implantation were shown to be more exsudative compared to the corresponding cases performed by AK. This could be related to the choice of the latex-based sheet that was placed on each reconstruction prior to Fixomull®-Stretch dressing pressure application. Nevertheless, the end result concerning healing behaviour and integration with the surrounding tissue along with the cosmetic outcome showed no differences between the individual surgeons. When looking at the large defect on the posterior scalp of case no. five, the healing time period was prolonged on account of a subsided wound surface. However, the defect was eventually completely filled up to the level of normal skin. This finding, which needs corroboration through further clinical data, is unexcepted, since it points to a possible volume-retaining or -inducing capacity of the BPCM when applied in multiple layers even in deep lesions. Furthermore, it has to be investigated why lesion sizes greater than 2 cm result in a more shiny appearance. Although spectulative at present, this phenomenon could be associated with the re-epitheliazation process. Thus, as it was postulated in case no. five that the dense layer of BPCM might hinder early tissue ingrowth from the wound base, the BPCM in the next clinical application (case six) underwent multiple punches as a prophylactic measure. Indeed, in the latter case, the present of punches was associated with a relatively rapid tissue regeneration in the preauricular region after application of BPCM. These preliminary findings necessitate a systematic clinical approach to confirm and prove the benefits of punched BPCM in deep skin defects, as it might allow a faster tissue ingrowth between the aritifically made wholes whithin the BPCM.

In recent years, a number of clinical studies have been performed with BPCM, showing its suitability for tissue regeneration within the oral cavity. In their studies Sanz et al*.* and Jepsen et al*.* showed successful dental root coverage and expansion of keratinized tissue after application of the BPCM [Sanz et al. 2009; Jepsen et al. 2013]. On the other hand, a clinical study has been undertaken to assess the long-term outcome of vestibuloplasty, which in some respect is the corresponding wound situation to an open wound within the oral cavity [Schmitt et al. 2015]. In the vestibuloplasty scenario the application of the BPCM does appear to contribute to soft tissue regeneration by adapting to the underlying tissue with its spongy site, while allowing the surrounding tissue to build epithelium along the compact layer to close the defect. This phenomenon is exactly what occurs during skin regeneration when applying this matrix for extraoral defect reconstruction. The process of stepwise integration of the BPCM matrix by means of the spongy layer and infiltration of the compact layer by mononuclear cells without breakdown of the latter layer might be the basis of the „specific“integration of this bilayered collagen matrix. Thus, the dense layer of the BPCM imitates the papillary layer, whereas the spongy layer simulates the reticular layer of the dermis.

Previously published data about BPCM have shown that non-cross-linked collagen membranes/matrices do not require extensive vascularization for good tissue integration and moreover do not undergo a transmembranous vascularization for their successful contribution to tissue regeneration [Ghanaati et al. 2011; Ghanaati 2012]. Additionally, they highlight that well processed and non-crosslinked collagen-based materials solely induce mononucler cells but no multinucleated giant cells [Ghanaati et al. 2011; Ghanaati 2012]. In a further combined in vitro and in vivo study a gradual cell ingrowth into the BPCM was demonstrated [Willershausen et al. 2014]. Other in vivo studies have shown that the presence of multinucleated giant cells within the implantation bed of collagen membranes may contribute not only to enhanced vascularization but also to their disintegration [Barbeck et al. 2014a; Barbeck et al. 2014b]. These data stress the need to re-evaluate the terminology “transmembranous vascularization”, which indicates a communication between the two tissue layers above and below the collagen-based material. However, this is only possible via biomaterial degradation.

A review of the histological data in the present case series underlines the fact that BPCM becomes integrated into the defect region without material breakdown. Moreover, BPCM is gradually penetrated by mononuclear cells in the form of mainly TRAP-negative macrophages. These findings suggest that BPCM integration into the subepidermal connective tissue takes place without evoking a severe inflammatory reaction or biomaterial degradation. Moreover, the spongy site of the matrix seems to serve as a „replacer“of the lost connective tissue and allows a stepwise cell and connective tissue ingrowth from the wound-material-interface towards the central region of the matrix. Furthermore, it is conceivable that the compact layer promotes a mononuclear cell penetration, without major break down. This hypothesis can be supported by the findings of a recently performed animal study, in which the cell penetration of the compact layer of the BPCM took place within the first 14 days and was independent of the integration of the spongy layer [Ghanaati et al. 2011].

The most interesting aspect of the regenerative mechanism of this collagen matrix is the simultaneous epidermal regeneration. Interestingly, the histology revealed that a completely differentiated epidermal layer could be observed above the implantation area of the matrix in the case of small-sized skin defects at 14 days after implantation. In the case of middle-sized defects at least a thin epithelial layer within the central defect region was found, which matured with time. Thus, the collagen matrix contributes to the regeneration of both subepidermal connective tissue and epidermal tissue, presumably as a result of its bilayered design. The compact surface appears to act favourably as a membrane for epidermal regeneration from the edges, whilst the porous spongy layer acts as a scaffold for underlying soft tissue regeneration. In this context it is conjecturable that the bi-layered structure, i.e. the compact and the spongy side of the material have led to a new understanding of a “structure” dependent integration of collagen matrices. Thus care has to be given in speaking about GTR (Guided Tissue Regeneration), according to which a „barrier“membrane is needed. The present data and above mentioned preclinical data from our group showing distinct and more or less independent integration processes of the different parts of BPCM prompts us to look at biomaterial assisted tissue regeneration as a “structure guided whole tissue integrative process”. In this GIRP „(Guided-Integrative-Regeneration-Process) the spongy layer acts as the scaffolds especially for the regeneration of the connective tissue part of the skin, while the compact layer seems to serve as basis for the conductive growth of epidermis. It is evident that further molecular biological and immunhistological analyses are necessary in order to delineate the underlying regenerative mechanisms, for example, the relationship between the mononuclear cell penetration of the compact layer and its ability to promote epidermis regeneration. Apparently, components within the extracellular matrix are contributing to the successful acceptance of the grafted materials and its guiding role in the skin remodeling process. It is probable that the latter is initiated and regulated by the contained matricellular proteins, as this protein family is known to be involved in intra- and intercellular communication and signaling, including control of growth factor-mediated actions, such as platelet-derived growth factor-mediated cell migration [Zhang et al. 2015]. This protein family is also known to be involved in the initiation of the inflammatory response, such as leukocyte trafficking [Emre and Imhof 2014]. Future possibilities include application of knowledge of the role of the matricellular proteins to direct the clinical outcome of the defects after biomaterial implantation. This could be achieved by specific functionalization of the material surfaces.

From a clinical translational point of view the present data open up new vistas on current „traditional“plastic surgical strategies for skin regeneration, including conservative surgery with the help of potent biomaterials to compensate for missing skin structures. The possibility of applying animal-based collagen matrices for full skin reconstruction paves the way for new concepts in reconstructive plastic surgery, especially as the application of biomaterials alone or in combination with free or regional flaps would significantly increase the chances for successful conservative reconstructive plastic surgery. Especially older people would benefit from such biomaterial-based reconstructive concepts, as their health condition is in many cases compromised due to multimorbidity. The present data, however, also expose the need for further clinical assessment of this matrix in systematic clinical studies, in which it should be compared with current gold standard regimens, namely split-skin grafts or free granulation.

## Conclusion

In the present clinical study a porcine bilayered non-cross-linked collagen matrix was used to reconstruct facial soft tissue defects of varying extent after skin cancer resection in nasal, eyelid, forehead and scalp regions. The defect size ranged from 1,2 to 6 cm in diameter. Wound closure in small defects by epidermis was achieved within a time period of 14 days, whereas complete regeneration required 4–5 weeks. In larger defects wound closure by the epidermis took longer and complete regeneration was achieved in 8–24 weeks. In all cases the newly regenerated skin was very similar to the neighbouring tissue in terms of coloration, structure, and consistency and showed no sign of visible scars or tension. The use of the bilayered matrix contributed to a complete tissue integrative process, i.e. the spongy site serves as a connective tissue guide and scaffold, while the compact layer serves as a membrane-like growth substratum for epithelial cells. The present data encourage re-thinking of the traditional flap-based plastic surgery for comparable defects. Beside these clinical and histological findings of the present study, knowledge concerning the mechanism underlying the integration of collagen based membranes/matrices and biomaterials in general is necessary to be able to design materials for specific tissue defects. The involvement of various matricellular proteins is certainly a rational starting point.

## References

[CR1] Anderson JM (2001). Biological responses to materials. Annu Rev Mater Res.

[CR2] Barbeck M, Lorenz J, Kubesch A, Booms P, Boehm N, Choukroun J, Sader R, Kirkpatrick CJ, Ghanaati S (2014) Porcine dermis-derived collagen membranes induce implantation bed vascularization via multinucleated giant cells: a physiological reaction? J Oral Implantol. doi:10.1563/aaid-joi-D-14-00274 10.1563/aaid-joi-D-14-00274 25546240

[CR3] Barbeck M, Lorenz J, Grosse Holthaus M, Raetscho N, Kubesch A, Booms P, Sader R, Kirkpatrick CJ, Ghanaati S (2014) Porcine dermis and pericardium-based, non cross-linked materials induce multinucleated giant cells after their in vivo implantation: A physiological reaction? J Oral Implantol. doi:10.1563/aaid-joi-D-14-00155 10.1563/aaid-joi-D-14-00155 25386662

[CR4] Bornstein P, Sage EH (2002). Matricellular proteins: extracellular modulators of cell function. Curr Opin Cell Biol.

[CR5] Cardaropoli D, Tamagnone L, Roffredo A, Gaveglio L (2012). Treatment of gingival recession defects using coronally advanced flap with a porcine collagen matrix compared to coronally advanced flap with connective tissue graft: a randomized controlled clinical trial. J Periodontol.

[CR6] Eickholz P, Krigar DM, Kim TS, Reitmeir P, Rawlinson A (2007). Stability of clinical and radiographic results after guided tissue regeneration in infrabony defects. J Periodontol.

[CR7] Emre Y, Imhof BA (2014). Matricellular protein CCN1/CYR61: a new player in inflammation and leukocyte trafficking. Semin Immunopathol.

[CR8] Ghanaati S. (2012). Non-cross-linked porcine-based collagen I-III membranes do not require high vascularization rates for their integration within the implantation bed: a paradigm shift. Acta Biomater.

[CR9] Ghanaati S, Schlee M, Webber MJ, Willershausen I, Barbeck M, Balic E, Görlach C, Stupp SI, Sader RA, Kirkpatrick CJ (2011). Evaluation of the tissue reaction to a new bilayered collagen matrix in vivo and its translation to the clinic. Biomed Mater.

[CR10] Gottlow J, Nyman S, Karring T, Lindhe J (1984). New attachment formation as the result of controlled tissue regeneration. J Clin Periodontol.

[CR11] Herford AS, Akin L, Cicciu M, Maiorana C, Boyne PJ (2010). Use of a porcine collagen matrix as an alternative to autogenous tissue for grafting oral soft tissue defects. J Oral Maxillofac Surg.

[CR12] Jepsen K, Jepsen S, Zucchelli G, Stefanini M, de Sanctis M, Baldini N, Greven B, Heinz B, Wennström J, Cassel B, Vignoletti F, Sanz M (2013). Treatment of gingival recession defects with a coronally advanced flap and a xenogeneic collagen matrix: a multicenter randomized clinical trial. J Clin Periodontol.

[CR13] Kyriakides TR, Bornstein P (2003). Matricellular proteins as modulators of wound healing and the foreign body response. Thromb Haemost.

[CR14] Lorenz J, Kubesch A, Korzinskas T, Barbeck M, Landes C, Sader R, Kirkpatrick CJ, Ghanaati S (2014) TRAP-positive multinucleated giant cells are foreign body giant cells rather than osteoclasts: Results from a split-mouth study in humans. J Oral Implantol. doi:10.1563/aaid-joi-D-14-00273 10.1563/aaid-joi-D-14-00273 25490579

[CR15] Morris AH, Kyriakides TR (2014). Matricellular proteins and biomaterials. Matrix Biol.

[CR16] Pretzl B, Kim TS, Holle R, Eickholz P (2008). Long-term results of guided tissue regeneration therapy with non-resorbable and bioabsorbable barriers. IV. A case series of infrabony defects after 10 years. J. Periodontol.

[CR17] Rogers-Vizena CR, Lalonde DH, Menick FJ, Bentz ML (2015). Surgical treatment and reconstruction of nonmelanoma facial skin cancers. Plast Reconstr Surg.

[CR18] Sameem M, Au M, Wood T, Farrokhyar F, Mahoney J (2012). A systematic review of complication and recurrence rates of musculocutaneous, fasciocutaneous, and perforator-based flaps for treatment of pressure sores.. Plast Reconstr Surg.

[CR19] Sandu K, Monnier P, Pasche P (2012). Supraclavicular flap in head and neck reconstruction: experience in 50 consecutive patients. Eur Arch Otorhinolaryngol.

[CR20] Sanz M, Lorenzo R, Aranda JJ, Martin C, Orsini M (2009). Clinical evaluation of a new collagen matrix (mucograft prototype) to enhance the width of keratinized tissue in patients with fixed prosthetic restorations: a randomized prospective clinical trial. J Clin Periodontol.

[CR21] Schmitt CM, Tudor C, Kiener K, Wehrhan F, Schmitt J, Eitner S, Agaimy A, Schlegel KA (2013). Vestibuloplasty: porcine collagen matrix versus free gingival graft: a clinical and histologic study. J Periodontol.

[CR22] Schmitt CM, Moest T, Lutz R, Wehrhan F, Neukam FW, Schlegel KA (2015). Long-term outcomes after vestibuloplasty with a porcine collagen matrix (mucograft(®)) versus the free gingival graft: a comparative prospective clinical trial. Clin Oral Implants Res.

[CR23] Willershausen I, Barbeck M, Boehm N, Sader R, Willershausen B, Kirkpatrick CJ, Ghanaati S (2014). Non-cross-linked collagen type I/III materials enhance cell proliferation: in vitro and in vivo evidence. J Appl Oral Sci.

[CR24] Zhang F, Hao F, An D, Zeng L, Wang Y, Xu X, Cui MZ (2015). (2015). the matricellular protein Cyr61 is a key mediator of platelet-derived growth factor-induced cell migration. J Biol Chem.

[CR25] Zhao MX, Li YQ, Tang Y, Chen W, Yang Z, Hu CM, Liu YY, Xu LS (2012). Infraorbital and zygomatic reconstruction using pre-expanded rotation flap based on the orbicularis oculi muscle. J Plast Reconstr Aesthet Surg.

